# Nutrient Deprivation-Associated Changes in Green Microalga *Coelastrum* sp. TISTR 9501RE Enhanced Potent Antioxidant Carotenoids

**DOI:** 10.3390/md17060328

**Published:** 2019-06-01

**Authors:** Monrawat Rauytanapanit, Kantima Janchot, Pokchut Kusolkumbot, Sophon Sirisattha, Rungaroon Waditee-Sirisattha, Thanit Praneenararat

**Affiliations:** 1 The Chemical Approaches for Food Applications Research Group, Faculty of Science, Chulalongkorn University, Phayathai Rd., Pathumwan, Bangkok 10330, Thailand; monrawat.mon@gmail.com (M.R.); kantima.janchot@gmail.com (K.J.); rungaroon.w@chula.ac.th (R.W.-S.); 2 Department of Chemistry, Faculty of Science, Chulalongkorn University, Phayathai Rd., Pathumwan, Bangkok 10330, Thailand; 3 Department of Microbiology, Faculty of Science, Chulalongkorn University, Phayathai Rd., Pathumwan, Bangkok 10330, Thailand; 4 Thailand Institute of Scientific and Technological Research (TISTR), Khlong Luang, Pathum Thani 12120, Thailand; pokchut@tistr.or.th (P.K.); sophon_si@tistr.or.th (S.S.)

**Keywords:** *Coelastrum*, microalgae, astaxanthin, canthaxanthin, lutein, carotenoids

## Abstract

The utilization of microalgae as a source of carotenoid productions has gained increasing popularity due to its advantages, such as a relatively fast turnaround time. In this study, a newly discovered *Coelastrum* sp. TISTR 9501RE was characterized and investigated for its taxonomical identity and carotenoid profile. To the best of our knowledge, this report was the first to fully investigate the carotenoid profiles in a microalga of the genus *Coelastrum*. Upon use of limited nutrients as a stress condition, the strain was able to produce astaxanthin, canthaxanthin, and lutein, as the major carotenoid components. Additionally, the carotenoid esters were found to be all astaxanthin derivatives, and β-carotene was not significantly present under this stress condition. Importantly, we also demonstrated that this practical stress condition could be combined with simple growing factors, such as ambient sunlight and temperature, to achieve even more focused carotenoid profiles, i.e., increased overall amounts of the aforementioned carotenoids with fewer minor components and chlorophylls. In addition, this green microalga was capable of tolerating a wide range of salinity. Therefore, this study paved the way for more investigations and developments on this fascinating strain, which will be reported in due course.

## 1. Introduction

**Figure 1:**
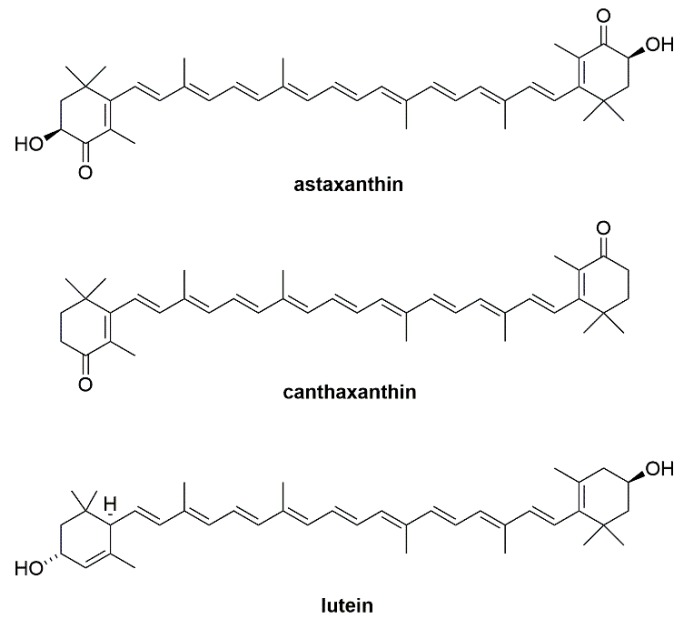
Chemical structures of important carotenoids with various applications.

Carotenoids are an important class of natural tetraterpenes found in several plants, algae, fungi, and bacteria [1,2,3,4,5]. To date, there are several hundred characterized carotenoids found in various sources, some of which were found to play important roles in their respective organisms. Examples include being an integral part of the photosynthesis unit [6,7], or playing photoprotective roles [8,9,10]. Interestingly, the discovery of these aforementioned roles simultaneously inspired the investigation of these compounds for use in other applications. Consequently, several carotenoids, some of which are depicted in Figure 1, have found uses in a number of areas [11,12,13,14]. For instance, astaxanthin was found to be the strongest antioxidant carotenoid in nature, with power that was several-fold stronger than that of β-carotene [4,15,16]. Thus, it is currently in high demand for use in the nutraceutical, pharmaceutical, and cosmetic industries [17,18,19,20,21]. Another example is canthaxanthin, which was found to serve as a food colorant in animal feed for several food sources, such as poultry and aquatic animals [22,23,24]. Lastly, lutein has been demonstrated to exhibit crucial roles in human eye health [25,26,27]. Clearly, these attractive properties of carotenoids have led to an increasing demand for them, and thus the development of economic, high-yielding productions of carotenoids are continuously being studied [4,5,28,29,30,31,32].

Despite the fact that the chemical syntheses of some carotenoids are available, these methods have limited utilization in real applications owing to health concerns arising from the use of pure compounds instead of isomeric mixtures that typically exist in natural sources [4,33]. Hence, naturally obtained carotenoids are usually preferred, leading to the research and development of efficient carotenoid extraction methods from natural sources [4,5,31]. Nevertheless, except for some cases like lutein extraction from Marigold [34,35], microalgal sources have become more important than plants as a source of carotenoids [2,4,13,29]. This is due to the fact that microalgae possess a desirable balance between being autotrophs capable of producing a range of carotenoids, and having biotechnological-related advantages, such as fast growth and easier genetic manipulation. Prominent examples include astaxanthin from *Haematococcus pluvialis* and *Xanthophyllomyces dendrorhous*, and β-carotene from *Dunaliella salina*, both of which have been utilized in actual commercial production [36,37,38].

Notably, some enhancement cues are typically required for substantial production of the carotenoids of interest, which is usually in the form of stress conditions. Heat, light, high salt concentrations, and depleted nitrogen supply are common stress conditions used to induce carotenoid production in microalgae [30,39,40,41,42,43]. Interestingly, *Coelastrum* is a genus of green algae that was also found to be capable of producing carotenoids, although there have been few reports that investigated the carotenoid profiles of this genus. *Coelastrum* sp. HA-1, an isolated microalga from Bohai Bay, China, was cultivated under nitrogen-limiting conditions and it produced astaxanthin at a level of 6.36 mg/g dried cell weight [44]. The cultivation of *Coelastrum* cf. *pseudomicroporum* in urban wastewater under salt stress conditions produced carotenoids at a level of 33.4 ± 19.86 pg cell^−1^ [45]. Lastly, Soares et al. reported that *Coelastrum sphaericum* provided a set of carotenoids with the prominent components being astaxanthin and lutein [46]. 

In this study, a strain of *Coelastrum* sp. was isolated and investigated for its ability to enhance carotenoid production under stress conditions. The induction condition was a drastic reduction of the nutrient contents, which could also be viewed as a practical advantage in a real production process (cost reduction). Notably, in contrast to the aforementioned studies [44,45], this study serves as the first example of this genus, where the carotenoid profile is characterized by both HPLC-PDA (photodiode array detection) and LC-MS. Molecular identification of this microalgal strain, its growth profiles, and the carotenoid identifications and quantifications are discussed herein.

## 2. Results and Discussion

### 2.1. Morphology and Genetic Identification of the Microalga Coelastrum sp. TISTR 9501RE

**Figure 2:**
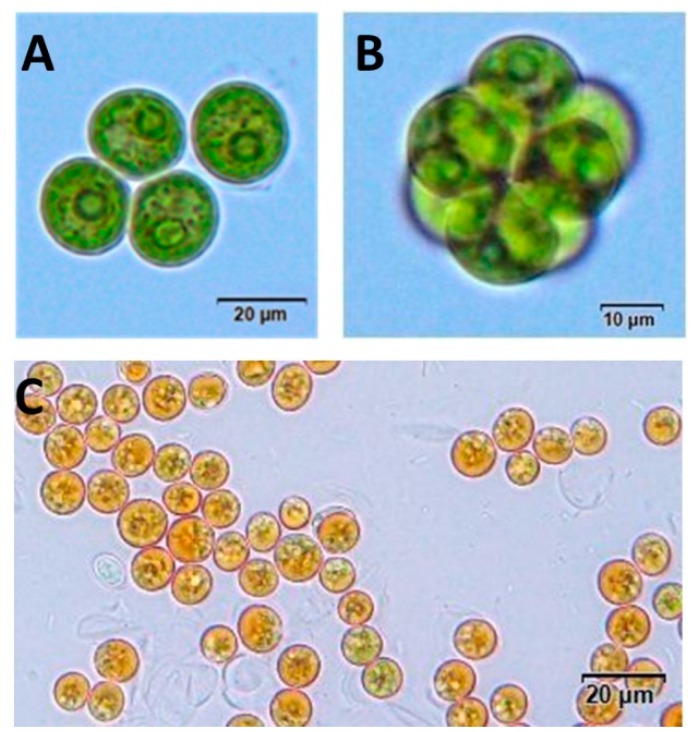
Light microscopic images of *Coelastrum* sp. TISTR 9501RE; (**A**) Green vegetative cells; (**B**) A spherical colony; (**C**) Carotenoids accumulating cells.

Isolated from a coastal ecosystem in the northern part of Thailand, this strain was discovered from the screening of strains that are capable of enhancing carotenoid accumulations upon the stress conditions of interest. In this regard, we employed a nutrient-depleted condition to induce the carotenoid biosynthesis. Notably, the condition used was an overall reduction of the required nutrient (BG11), such that it was only one-fourth of the normal formula. This provided the added benefit of drastically reducing the overall production costs for larger scale production. Apparently, this led to an obvious concern regarding whether this reduced nutrient had overly affected the growth of the microalga. Hence, we sought to observe the growth rates under both conditions. The results (Figure S1) showed that the growth, albeit being unsurprisingly suppressed, could still reach OD_730_ at around 1.0. Compared to other studies [44], this level of cell mass was at an acceptable level. Prior to the induction condition, the algal colonies on the BG11 agar exhibited a dull-shiny texture, olive green color, and circular shape. Microscopic morphology observations showed that the algal cells were spherical vegetative cells varying between 8 and 15 µm in diameter (Figure 2A). The colonies were also spherical (Figure 2B). After 14 days of growth on one-fourth BG11 agar, there was an obvious accumulation of a bright orange color, which we hypothesized to be carotenoid-based pigments (Figure 2C). Furthermore, we also demonstrated that this microalga was capable of tolerating a wide range of salinities (up to the relevant concentration in the ocean at 500 mM NaCl) (Figure S2), thereby paving the way for more diverse applications. Given its spherical shape, this algal strain was preliminarily hypothesized to belong to the genus Coelastrum [47].

**Figure 3:**
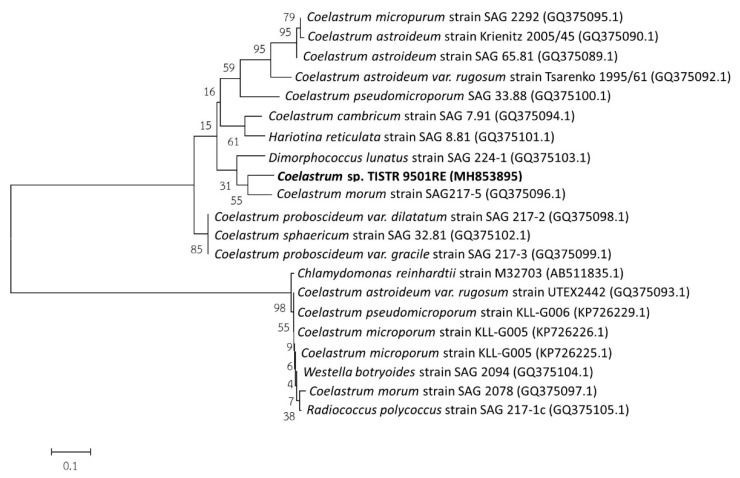
Maximum likelihood (ML) tree from the sequence-structure analysis of the ITS sequence data. The tree was generated by the neighbor joining method using the Molecular Evolutionary Genetic Analysis (MEGA6) software. The bootstrap value is expressed as a percentage of 100 replicates. The nucleotide sequence accession numbers are indicated in parentheses.

To better confirm its identity, standard genetic identification was accomplished using genomic sequencing of the internal transcribed spacer (ITS) 1 region of the 5.8S ribosomal RNA gene. To verify its taxonomical position, the ITS1 of the 5.8S rRNA gene was compared to sequences in public databases (e.g., GenBank). Similar sequences were used to construct independent molecular phylogenetic trees based on the ITS1-5.8s-ITS2 sequence. The reliability of the phylogenetic tree was evaluated using neighbor joining analysis. As shown in Figure 3, the ITS1-5.8s-ITS2 sequence comparison revealed that strain TISTR 9501RE was grouped together with other *Coelastrum* species in the same clade, thereby confirming that this strain was a species in the genus Coelastrum. Based on both the morphological and molecular evidence, this microalga was named *Coelastrum* sp. TISTR 9501RE, which is a member of the green algae (Chlorophyta).

### 2.2. Carotenoid Profiles of Coelastrum sp. TISTR 9501RE

To characterize the molecular components of the putative carotenoid mixture observed from the morphological change of the cell, an extraction procedure by bead-beating [4,31] was employed for both the control (normal nutrient strength) condition and the nutrient-depleted condition. Thereafter, the obtained pigment mixtures were subjected to total chlorophyll determination via UV-vis spectroscopy. This revealed that the nutrient-depleted condition resulted in a significant decrease in chlorophyll production in the *Coelastrum* sp. TISTR 9501RE. That is, the total chlorophyll content substantially decreased from 5.64 ± 0.12 mg of total chlorophylls per one gram of dried weight of the cells (mg/g DW) to 3.89 ± 0.03 mg/g DW when the nutrient amount was restricted. This could also be illustrated by calculation of the chlorophyll a and b contents, which clearly showed that the amounts of both compounds were significantly reduced (chlorophyll a: 5.08 ± 0.10 to 3.37 ± 0.02 mg/g DW; chlorophyll b: 1.09 ± 0.02 to 0.92 ± 0.01 mg/g DW). Nevertheless, these numbers merely served as a rough guideline, and the identifications of the specific types of compounds were deemed to be more important.

**Figure 4:**
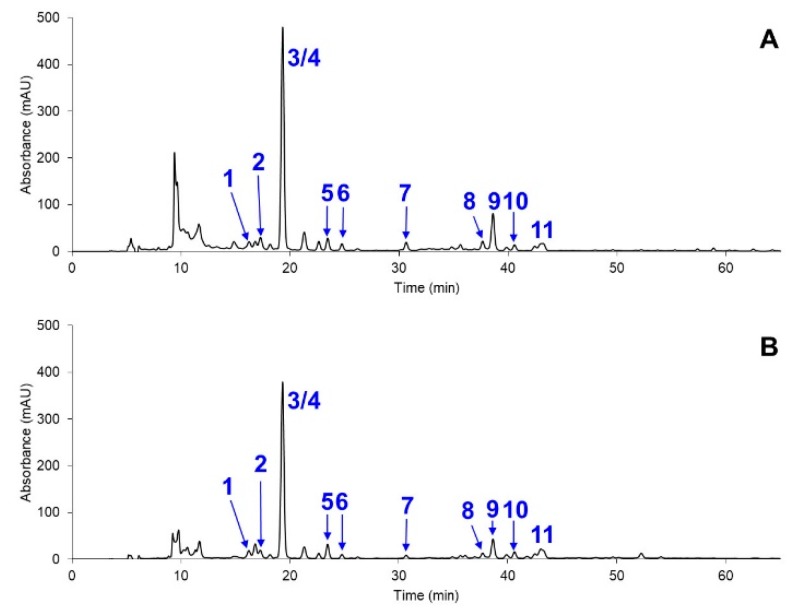
Chromatograms (representative data from three experiments at 450 nm) of the carotenoid extract from *Coelastrum* sp. TISTR 9501RE, with (**A**) the control condition, and (**B**) the nutrient depleted condition. Identities of the peaks can be found in Table 1 and Table 2.

To gain more insight on the composition of the carotenoid mixtures, high performance liquid chromatography with photodiode array detection (HPLC-PDA) and liquid chromatography–mass spectrometry (LC-MS) experiments were conducted. These data, along with some comparisons from previous literature [48], resulted in the identification of the carotenoids of interest; most of which are shown in Figure 4, and Table 1; Table 2. Overall, three carotenoids of interest, namely astaxanthin (16.2 min), lutein (19.3 min, co-eluted with chlorophyll b), and canthaxanthin (23.5 min), were produced in appreciable amounts in this strain. Other prominent peaks included chlorophylls and carotenoid esters (putatively derived from astaxanthin), along with some unidentified steroid and glyceride species (as suggested by MS data not shown) scattered throughout the chromatograms. Compared to the control conditions, the nutrient-depleted microalgae showed discernible changes in the carotenoid biosynthesis. For instance, the syntheses of the unidentified steroid-related species around 10 min were suppressed, and the amounts of all the chlorophyll-related species, e.g., peak 6 and 9, were significantly decreased. On the other hand, the syntheses of all the aforementioned carotenoids were increased, as quantified by the LC-MS (see below). This suggested that the astaxanthin biosynthesis pathway could be legitimately up-regulated, as canthaxanthin was also produced in a significant amount. This was in-line with the pathway of *H. pluvialis* [49], where echinenone and canthaxanthin were direct precursors to astaxanthin. Furthermore, it seemed that fatty acid esters, which were commonly found in several organisms, were all derivatives of astaxanthin. Although many of them were present in minute amounts (there were in fact some additional astaxanthin esters in small but MS-detectable amounts that are not shown in Table 1 and Table 2), the combined amounts of all the esters could be considered as a significant addition to the free form, thereby confirming the enhanced production of astaxanthin.

Thereafter, some quantitative studies were conducted to allow for further comparisons with previous studies. In this regard, we prepared calibration plots (Figure S3) and quantified three compounds, namely astaxanthin, canthaxanthin, and lutein, using LC-MS experiments. This provided the benefit of not having to develop a new method, since certain compounds were co-eluted with other components, thereby causing some quantification errors if HPLC-PDA were to be used. As a result, we determined that the strain *Coelastrum* sp. TISTR 9501RE produced all the compounds in higher amounts when stressed with limited nutrients (Table 3). However, as alluded above, the total astaxanthin was higher owing to the conjugation with various fatty acids.

Interestingly, when compared to previous studies, the *Coelastrum* sp. TISTR 9501RE exhibited a unique carotenoid profile. For example, our strain yielded about one half of the amount of all-trans-astaxanthin as did *H. pluvialis* from Jin and coworkers (0.25 ± 0.04 mg/g DW vs. 0.11 ± 0.01 mg/g DW in our case) under that study’s nitrogen deficiency condition [50]. However, none of their conditions, including conditions with higher astaxanthin contents, yielded comparable amounts of canthaxanthin and lutein as obtained in our case. On the other hand, a dark condition from their study did provide a significant amount of lutein, but at the expense of the total absence of astaxanthin and canthaxanthin. Significantly higher amounts of astaxanthin could be obtained, although with other stress conditions requiring high energy, such as 6000-lx cool white fluorescent light. Interestingly, whilst *H. pluvialis* is well known as the most efficient astaxanthin producer, it has some notable drawbacks, including slow growth at room temperature, ease of contamination by other microalgae, and a high light requirement [51]. Hence, it is relatively uncommon to cultivate this strain in more relaxed, but large-scale conditions, such as the one demonstrated herein (see below), which has prompted researchers to find alternative species [52]. For more similar organisms, a report by Liu et al. [44] showed that *Coelastrum* sp. HA-1 could produce astaxanthin at 6.36 mg/g DW, although it was not possible to compare it with other carotenoid profiles, since there was no identification of other carotenoids at all. Similarly, the work by Minhas et al. reported the production of 1.17 mg/L of astaxanthin and 0.64 mg/g DW of lutein for *Coelastrella* sp. (P63), but there was no information on other carotenoid species [53]. Importantly, the currently reported *Coelastrum* sp. TISTR 9501RE produced a relatively limited amount of β-carotene, and only under the control condition (around 62.5 min in Figure 4A), which was in contrast to some of the aforementioned works. Another example is from Hu and coworkers [54], who reported the presence of five components, including astaxanthin, canthaxanthin, lutein, β-carotene, and adonirubin, without the identification of other carotenoids. 

**Figure 5:**
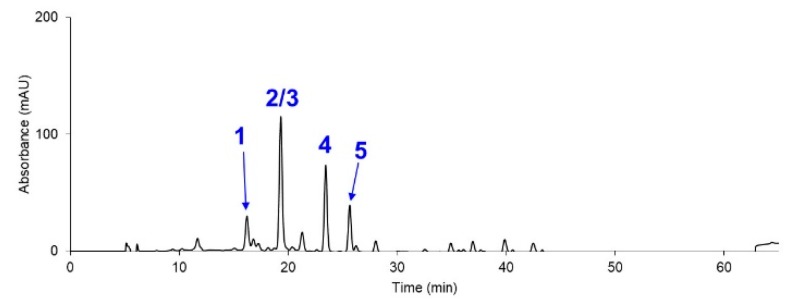
Chromatogram data (450 nm) of the carotenoid extract from the *Coelastrum* sp. TISTR 9501RE in a 20,000-L open raceway pond. Identities of the peaks can be found in Table 4.

Last but not least, since the stress condition used in this study provided the practical advantage of consuming less nutrients, we explored the possibility of growing this microalga on a much larger scale. To demonstrate its great applicability, we employed ambient sunlight, ambient temperature (ranging from 30 to 35 °C), groundwater as the water source, paddle wheels for stirring without active air feeding, and the same reduced nutrient (1/4 diluted BG11) to cultivate the *Coelastrum* sp. TISTR 9501RE in a 20,000-L open raceway pond. Interestingly, the HPLC and LC-MS analyses (Figure 5 and Table 4) of the extract showed a drastically simpler carotenoid profile, where all the major peaks were only astaxanthin, lutein, and canthaxanthin (excluding two chlorophyll species). Given that these carotenoids are attractive candidates for nutraceutical applications [21] due to their potent antioxidant activities, this condition thereby served as a prime example for more extensive application in large-scale productions in the future.

## 3. Materials and Methods

### 3.1. Microalgal Strain and Culture Conditions

The green microalga *Coelastrum* sp. was isolated from a coastal ecosystem in northern Thailand (obtained from the algae library of the Thailand Institute of Scientific and Technological Research (TISTR)). Cells were grown photoautotrophically (70 µmol m^−2^ s^−1^) in BG11 medium [55] or onto BG11 agar at 25 °C, unless otherwise stated. For carotenoids induction, the algal cells were inoculated at 3% (*v/v*) into 50 mL of BG11 and one-fourth strength BG11 medium (three replicates). All the flasks were shaken at 110 rpm under light 75–100 µmol m^−2^ s^−1^ for 14 days, with intermittent OD_730_ measurements for growth study (which was repeated for three independent experiments). The cells were harvested using centrifugation (12,000 × *g*, 10 min, 4 °C), and then dehydrated using a Flexi-Dry MP freeze dryer (Kinetics, Stone Ridge, NY, USA). The freeze-dried cells were used for carotenoids extraction (see below), and the subsequent HPLC analyses. The effect of salinity on growth of the microalgal strain was performed onto BG11 agar supplemented with different doses of NaCl (0, 0.15, 0.30, 0.40, and 0.5 M).

For large-scale production, the algal cells (10% inoculum) were cultivated in a 20,000-L open raceway pond equipped with paddle wheels (to ensure thorough mixing) under natural sunlight in one-fourth strength BG11 medium for 14 days. Cells were harvested by precipitation and centrifugation, using a SSE80-06-077 centrifuge (at 6200 rpm as per the manufacturer’s instructions, GEA Westfalia Separator Group GmbH, Oelde, Germany), followed by freeze drying and storage at −20 °C before analysis.

### 3.2. Molecular Identification and Polyphasic Taxonomy Approaches

All molecular cloning methods were performed according to standard protocols described in Reference [56]. PCR amplification of the internal transcribed spacer (ITS) 1 of the 5.8S ribosomal RNA gene was performed with the following primers: ITS forward 1 5′-TCCGTAGGTGAACCTGCGG-3′ and ITS reverse4 5′-TCCTCCGCTTATTGATATGC-3′. DNA sequencing was performed using an ABI 310 Genetic Analyzer (Applied Biosystems, Foster City, CA, USA). The ITS1-5.8s-ITS2 sequence was deposited into the GenBank under accession number MH853895. The phylogenetic tree was constructed and analyzed by the neighbor-joining method using the Molecular Evolutionary Genetics Analysis (MEGA6) software (free of charge from http://www.megasoftware.net). The robustness of the tree was assessed using bootstrap analysis (100 replicates).

### 3.3. Carotenoids Extraction

Thereafter, 70-mg freeze-dried cell powder was dissolved in 1-mL acetone and mixed with 0.5-mm silica glass beads (BeadBug™, Sigma-Aldrich, St. Louis, MO, USA). The resulting suspension was subjected to alternating cycles of vortexing and sonication as described below.

1. The suspension was vortexed for 5 min, followed by 2 min centrifugation at 10,000 rpm. The supernatant was collected, and 1 mL of fresh acetone was added to the crude precipitate. This process was repeated one more time.
2. The suspension from step 1 was sonicated for 30 min, followed by 2 min centrifugation at 10,000 rpm. Then, the supernatant was collected, and 1 mL of fresh acetone was added to the crude precipitate.
3. Step 1 was repeated exactly as shown above.
4. Step 2 was repeated exactly as shown above.
5. Step 1 was repeated exactly as shown above.

Then, all the supernatant fractions were combined. Thereafter, the solvent from the resulting solution was removed using a rotary evaporator. After that, the dried pigment mixture was ready for further analyses.

### 3.4. Determination of the Overall Chlorophyll Content

The overall content of chlorophylls was determined by adapting the method from Ritchie [57]. Briefly, crude extract was added with acetone to create a solution at a concentration of 0.5 mg/mL. This solution was then subjected to absorption measurement at 630, 647, 664, and 691 nm using a Cary 100 Bio-UV visible spectrophotometer (Agilent, Santa Clara, CA, USA). The resulting absorbance data were used to calculate the content of chlorophylls and carotenoids based on the following formulae (as µg/mL) [57].

Chlorophyll a = −0.3319A_630_ − 1.7485A_647_ + 11.9442A_664_ − 1.4306A_691_

Chlorophyll b = −1.2825A_630_ + 19.8839A_647_ − 4.8860A_664_ − 2.3416A_691_

Total Chlorophyll = 21.3877A_630_ + 10.3739A_647_ + 5.3805A_664_ + 5.5309A_691_

### 3.5. HPLC and LC-MS Analyses

A high performance liquid chromatograph (HPLC) with a photodiode array (PDA) detector was used to reveal the composition of the carotenoid mixtures. The following parameters were used in these experiments. Three separate experiments were performed for each sample, although one representative set was selected for illustration purposes in the results and discussion section.

Model: UltiMate 3000 HPLC ThermoFisher Scientific (Waltham, MA, USA); column: YMC30 reverse phase column (3 µm, ID 4.6 mm × 150 mm) (Kyoto, Japan); column temperature: 35 °C; injection volume: 10 µL; flow rate: 0.3 mL/min; mobile phase A: MeOH:MTBE (methyl *t*-butyl ether):H_2_O (81:15:4); mobile phase B: MeOH:MTBE:H_2_O (16:80.4:3.6). The time program can be found in Table S1 in the supplementary material.

Liquid chromatography–mass spectrometry (LC-MS) was performed to further characterize the carotenoids found in the mixture with the following parameters. Three separate experiments were performed for each sample, although one representative set was selected for illustration purposes in the results and discussion section.

Model: HPLC—ExionLC™ AD ultra-high performance liquid chromatograph (UHPLC); MS—SciEx X500R quadrupole time-of-flight MS (QTOF) (Framingham, MA, USA). MS parameters: mass range = 500–1300 *m/z*, positive mode; ion source gas 1 = 50 psi; ion source gas 2 = 50 psi; source temperature = 500 °C; spray voltage = 5500 V; declustering potential (DP) = 50 V; collision energy (CE) = 10 V.

The time programs for both the HPLC-PDA and LC-MS analyses were the same (Table S1). The concentrations of the mixtures used for the analysis were 10 and 2 mg/mL in acetone for the HPLC-PDA and LC-MS experiments, respectively. For the quantification experiments, standard solutions of the carotenoids of interest were prepared (500 ppm in acetonitrile for astaxanthin and canthaxanthin; 1000 ppm in acetone for lutein). Then each solution was serially diluted to different ranges that covered the relevant concentrations in the samples (0.5–8 ppm for astaxanthin, 1–20 ppm for canthaxanthin, and 10–150 ppm for lutein). All of these solutions were then analyzed and quantified by LC-MS in triplicates.

## 4. Conclusions

In conclusion, in this study, we reported the genetic identification and the study of carotenoid profiles of a new *Coelastrum* strain named *Coelastrum* sp. TISTR 9501RE. With the stress condition being the overall reduction in nutrients, the strain provided unique carotenoid compositions, with astaxanthin, canthaxanthin, and lutein being the major components. Interestingly, large-scale production could also be achieved under sustainable conditions such as ambient light, with even higher amounts of the desirable carotenoids. Further studies on a variety of stress conditions, especially the use of saltwater as a stress condition, and their effects on the biosynthesis pathways of carotenoids are an ongoing investigation in our group, so as to gain a better understanding and achieve more efficient carotenoid production.

## Figures and Tables

**Table 1:** Carotenoid profiles based on the LC-MS data of the extract of the control conditions from *Coelastrum* sp. TISTR 9501RE (a representative set of data from three experiments).

Entry	Identity *	Retention Time **	Proposed Formula	Precursor Mass	Found at Mass	Mass Error (ppm)
**1**	All-*trans*-astaxanthin	16.24	C_40_H_52_O_4_	597.3938	597.3936	−0.48
**2**	Violaxanthin isomer	17.28	C_40_H_56_O_4_	601.4251	601.4234	−2.91
**3**	All-*trans*-lutein	19.32	C_40_H_56_O_2_	569.4353	569.4317	−6.34
**4**	Chlorophyll b	19.32	C_55_H_70_N_4_O_6_Mg	907.5219	907.5214	−0.45
**5**	All-*trans*-Canthaxanthin	23.45	C_40_H_52_O_2_	565.4040	565.4030	−1.75
**6**	Chlorophyll a	25.65	C_55_H_72_N_4_O_5_Mg	893.5426	893.5393	−3.66
**7**	AME C18:4 isomer	30.66	C_58_H_78_O_5_	855.5922	855.5877	−5.27
**8**	AME C18:1 isomer	37.67	C_58_H_84_O_5_	861.6392	861.6346	−5.29
**9**	Chlorophyll b epimer	38.62	C_55_H_70_N_4_O_6_Mg	907.5219	907.5294	8.32
**10**	AME C18:2 isomer	41.76	C_58_H_82_O_5_	859.6235	859.6211	−2.85
**11**	Chlorophyll a epimer	42.96	C_55_H_72_N_4_O_5_Mg	893.5426	893.5474	5.43

* AME = Astaxanthin monoester. C18:n indicates an unsaturated fatty acyl part, with n being the number of double bonds in the molecule. ** Data from the HPLC-PDA run.

**Table 2:** Carotenoid profiles based on the LC-MS data of the extract of the nutrient-depleted conditions from *Coelastrum* sp. TISTR 9501RE (a representative set of data from three experiments).

Entry	Identity *	Retention Time **	Proposed Formula	Precursor Mass	Found at Mass	Mass Error (ppm)
**1**	All-*trans*-astaxanthin	16.22	C_40_H_52_O_4_	597.3938	597.3935	−0.53
**2**	Violaxanthin isomer	17.28	C_40_H_56_O_4_	601.4251	601.4251	−0.04
**3**	All-*trans*-lutein	19.33	C_40_H_56_O_2_	569.4353	569.4327	−4.54
**4**	Chlorophyll b	19.33	C_55_H_70_N_4_O_6_Mg	907.5219	907.5218	−0.03
**5**	All-*trans*-Canthaxanthin	23.46	C_40_H_52_O_2_	565.4040	565.4035	−0.87
**6**	Chlorophyll a	25.65	C_55_H_72_N_4_O_5_Mg	893.5426	893.5425	−0.12
**7**	AME C18:4 isomer	30.67	C_58_H_78_O_5_	855.5922	855.5918	−0.51
**8**	AME C18:1 isomer	37.69	C_58_H_84_O_5_	861.6392	861.6392	0.02
**9**	Chlorophyll b epimer	38.64	C_55_H_70_N_4_O_6_Mg	907.5220	907.5329	12.2
**10**	AME C18:2 isomer	41.77	C_58_H_82_O_5_	859.6235	859.6231	−0.49
**11**	Chlorophyll a epimer	43.00	C_55_H_72_N_4_O_5_Mg	893.5426	893.5507	9.04

* AME = Astaxanthin monoester. C18:n indicates an unsaturated fatty acyl part, with n being the number of double bonds in the molecule. ** Data from the HPLC-PDA run.

**Table 3:** Quantitative data from the LC-MS of three carotenoids (astaxanthin, lutein, and canthaxanthin) produced from *Coelastrum* sp. TISTR 9501RE under control and nutrient-depleted conditions. Data shown are averaged from three experiments.

Compound	Amount (mg/g DW)		
	Control Condition	Nutrient-Depleted Condition	Nutrient-Depleted Condition (20,000-L Pond)
All-*trans*-astaxanthin	0.03 ± 0.001	0.11 ± 0.01	0.18 ± 0.004
All-*trans*-lutein	2.35 ± 0.05	4.18 ± 0.46	3.13 ± 0.07
All-*trans*-Canthaxanthin	0.27 ± 0.03	1.15 ± 0.10	1.37 ± 0.03

**Table 4:** Carotenoid profiles based on the LC-MS data of the extract of the nutrient-depleted condition from *Coelastrum* sp. TISTR 9501RE in a 20,000-L open raceway pond (a representative set of data from three experiments).

Entry	Identity	Retention Time *	Proposed Formula	Precursor Mass	Found at Mass	Mass Error (ppm)
**1**	All-*trans*-astaxanthin	16.21	C_40_H_52_O_4_	597.3938	597.3935	−0.60
**2**	All-*trans*-lutein	19.32	C_40_H_56_O_2_	569.4353	569.4351	−0.39
**3**	Chlorophyll b	19.32	C_55_H_70_N_4_O_6_Mg	907.5219	907.5227	0.88
**4**	All-*trans*-Canthaxanthin	23.44	C_40_H_52_O_2_	565.4040	565.4044	0.65
**5**	Chlorophyll a	25.64	C_55_H_72_N_4_O_5_Mg	893.5426	893.5434	0.95

* Data from the HPLC-PDA run.

## Supplementary Materials

The following are available online at https://www.mdpi.com/1660-3397/17/6/328/s1, Figure S1: Growth curves of *Coelastrum* sp. TISTR 9501RE under (A) the control condition, and (B) the nutrient-depleted condition. Data shown are averaged from three experiments, Figure S2: Effect of salinity on the growth and cell morphology of *Coelastrum* sp. TISTR 9501RE. (A) Effect of salinity on the growth of *Coelastrum* sp. TISTR 9501RE was performed onto BG11 agar supplemented with different doses of NaCl (0, 0.15, 0.30, 0.40, and 0.50 M). For each concentration, 20 µL of microalgal culture in BG11 (OD_730_ ~ 1) was streaked onto BG11 at an indicated NaCl concentration. Plate cultures were incubated photoautotrophically (70 µmol m^−2^ s^−1^) at 25 °C for 3 days. Salinity tolerance was scored by assessing the growth or lack of growth. (B) Light microscopic images of *Coelastrum* sp. TISTR 9501RE were grown in BG11 liquid medium supplemented with different concentrations of NaCl (0, 0.15, 0.30, 0.45 M). Microalgal culture in BG11 (OD_730_ ~ 1) was inoculated into new BG11 liquid medium at each indicated NaCl concentration. Incubation condition was performed in the same way as indicated in (A). The morphology of the cells was observed under a light microscope (Olympus BX51, Japan), Figure S3: Calibration plots for astaxanthin (top), canthaxanthin (middle), and lutein (bottom) quantifications. Data shown are averaged from three experiments, Table S1: The time program for the HPLC-PDA and LC-MS experiments.
