# Supplementary material for: Nutrient Deprivation-Associated Changes in Green Microalga Coelastrum sp. TISTR 9501RE Enhanced Potent Antioxidant Carotenoids
Source: Mar Drugs. 2019 Jun 1;17(6):328. doi: 10.3390/md17060328 (PMC6627699; doi:10.3390/md17060328)
Supplement: Supplementary file 1 [file marinedrugs-17-00328-s001.pdf]

Nutrient Deprivation-Associated Changes in *Coelastrum* sp. (Green Microalga) TISTR 9501RE Enhanced Potent Antioxidant Carotenoids

Monrawat Rauytanapanit,[a,b] Kantima Janchot,[a,c], Pokchut Kusolkumbot,[d] Sophon Sirisattha,[d] Rungaroon Waditee-Sirisattha[a,c] and Thanit Praneenararat[a,b]

[a]The Chemical Approaches for Food Applications Research Group, Faculty of Science, Chulalongkorn University, Phayathai Rd., Pathumwan, Bangkok, 10330, Thailand. Email: Thanit.P@chula.ac.th.

[b]Department of Chemistry, Faculty of Science, Chulalongkorn University, Phayathai Rd., Pathumwan, Bangkok, 10330, Thailand.

[c]Department of Microbiology, Faculty of Science, Chulalongkorn University, Phayathai Rd., Pathumwan, Bangkok, 10330, Thailand.

[d]Thailand Institute of Scientific and Technological Research (TISTR), Khlong Luang, Pathum Thani 12120, Thailand.

***Corresponding Author**

Email: Thanit.P@chula.ac.th. Tel: +66-2-218-7638

Table S1. The time program for HPLC-PDA and LC-MS experiments.

Mobile phase A: MeOH:MTBE:H_2O (81:15:4)

Mobile phase B: MeOH:MTBE:H_2O (16:80.4:3.6)

Time (min)	Percentage of mobile phase	
	%A	%B
0	99	1
30	66	34
35	66	34
50	44	56
55	44	56
70	22	78
75	0	100
80	99	1
85	99	1

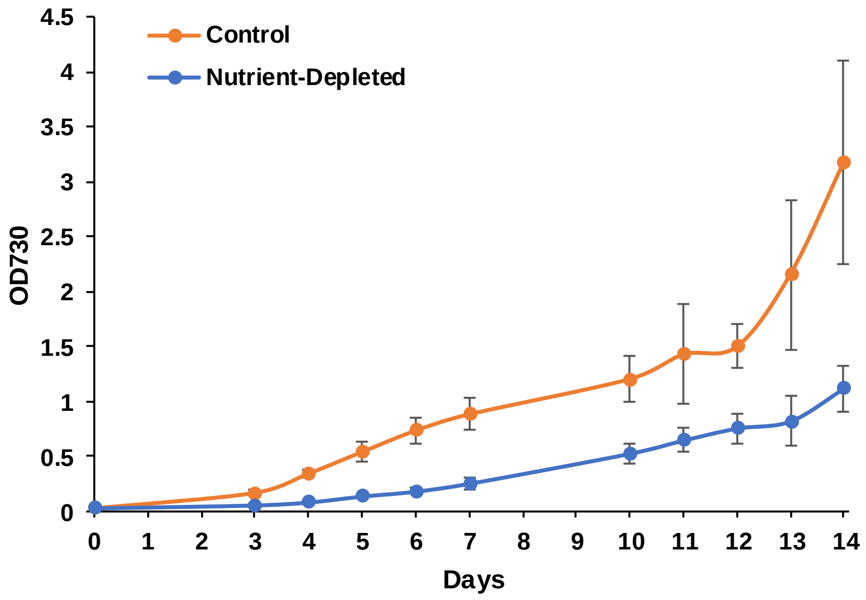

Figure S1. Growth curves of *Coelastrum* sp. TISTR 9501RE under A) control condition, and B) nutrient-depleted condition. Data shown are averaged from three experiments.

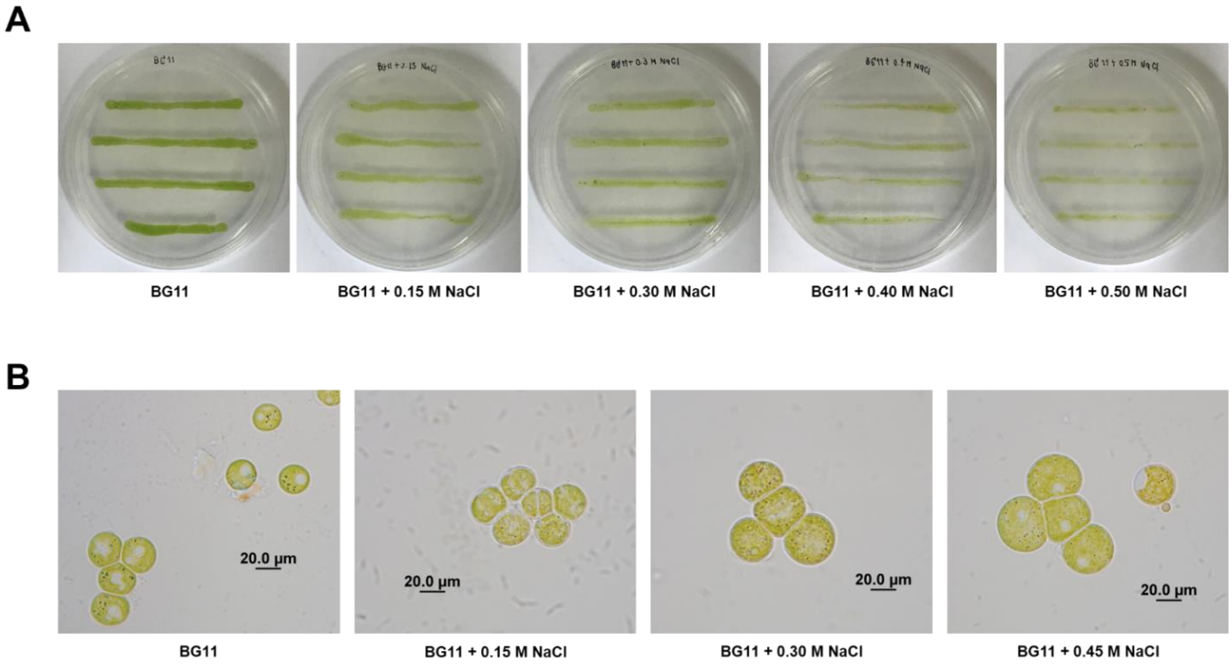

Figure S2. Effect of salinity on growth and cell morphology of *Coelastrum* sp. TISTR 9501RE. A) Effect of salinity on growth of *Coelastrum* sp. TISTR 9501RE was performed onto BG11 agar supplemented with different dose of NaCl (0, 0.15, 0.30, 0.40, and 0.50 M). For each concentration, 20 µL of microalgal culture in BG11 (OD_{730} ~ 1) was streaked onto BG11 at an indicated NaCl concentration. Plate cultures were incubated photoautotrophically (70 µmol m^{-2} s^{-1}) at 25 °C for 3 days. Salinity tolerance was scored by assessing growth or lack of growth. B) Light microscopic images of *Coelastrum* sp. TISTR 9501RE grown in BG11 liquid medium supplemented with different concentrations of NaCl (0, 0.15, 0.30, 0.45 M). Microalgal culture in BG11 (OD_{730} ~ 1) was inoculated into new BG11 liquid medium at each indicated NaCl concentration. Incubation condition was performed in the same way as indicated in A). The morphology of cells was observed under light microscope (Olympus BX51, Japan).

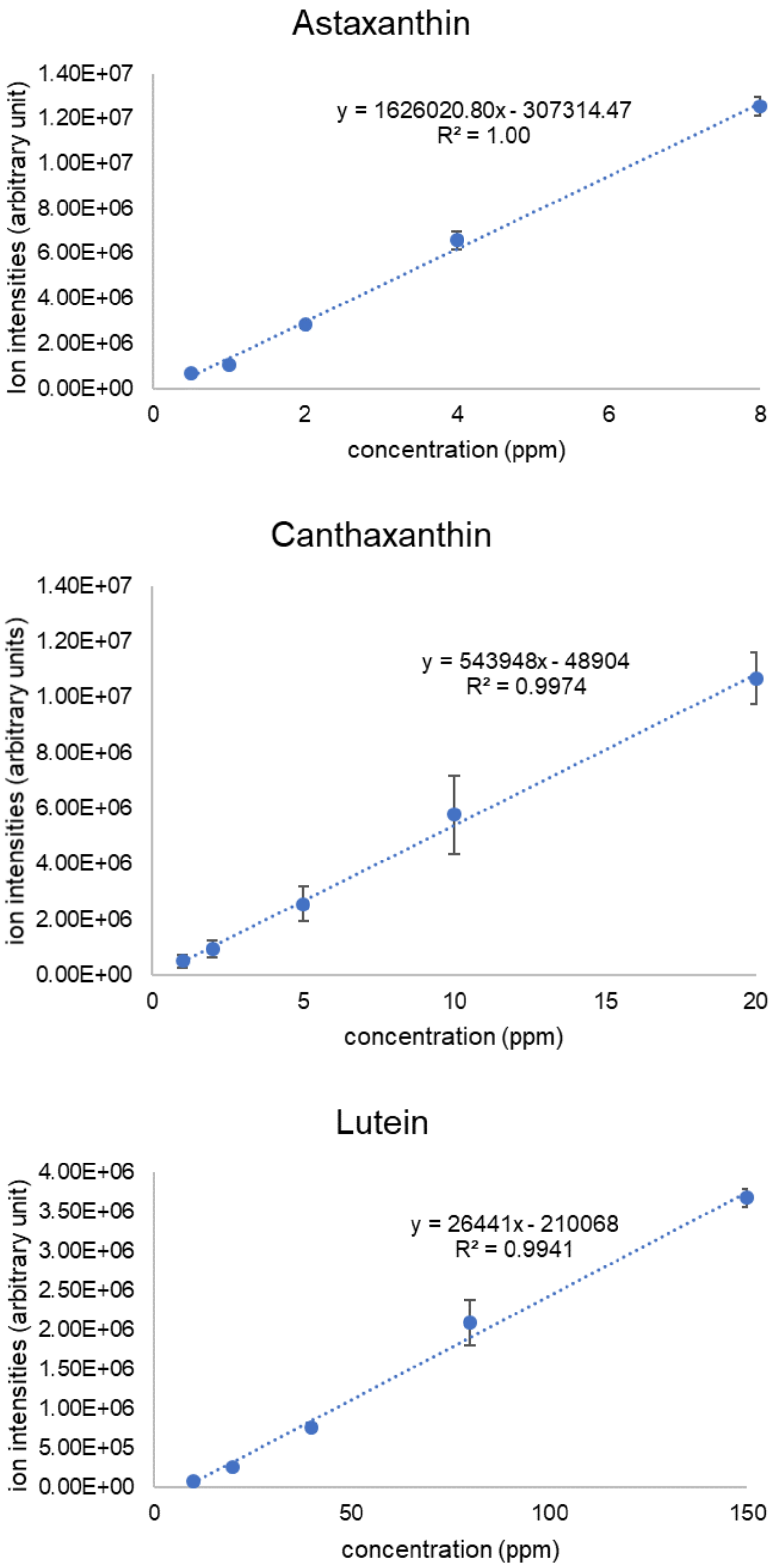

Figure S3. Calibration plots for astaxanthin (top), canthaxanthin (middle), and lutein (bottom) quantifications. Data shown are averaged from three experiments.
